# Computational Modeling Study of the Molecular Basis of dNTP Selectivity in Human Terminal Deoxynucleotidyltransferase

**DOI:** 10.3390/biom14080961

**Published:** 2024-08-07

**Authors:** Egor O. Ukladov, Timofey E. Tyugashev, Nikita A. Kuznetsov

**Affiliations:** 1 Institute of Chemical Biology and Fundamental Medicine, Siberian Branch of Russian Academy of Sciences, Novosibirsk 630090, Russia; e.ukladov@g.nsu.ru (E.O.U.); tyugashev@niboch.nsc.ru (T.E.T.); 2 Department of Natural Sciences, Novosibirsk State University, Novosibirsk 630090, Russia

**Keywords:** terminal deoxynucleotidyl transferase, TdT, enzyme, polymerase, dNTP, molecular dynamics, molecular modeling, rational design, DNA synthesis

## Abstract

Human terminal deoxynucleotidyl transferase (TdT) can catalyze template-independent DNA synthesis during the V(D)J recombination and DNA repair through nonhomologous end joining. The capacity for template-independent random addition of nucleotides to single-stranded DNA makes this polymerase useful in various molecular biological applications involving sequential stepwise synthesis of oligonucleotides using modified dNTP. Nonetheless, a serious limitation to the applications of this enzyme is strong selectivity of human TdT toward dNTPs in the order dGTP > dTTP ≈ dATP > dCTP. This study involved molecular dynamics to simulate a potential impact of amino acid substitutions on the enzyme’s selectivity toward dNTPs. It was found that the formation of stable hydrogen bonds between a nitrogenous base and amino acid residues at positions 395 and 456 is crucial for the preferences for dNTPs. A set of single-substitution and double-substitution mutants at these positions was analyzed by molecular dynamics simulations. The data revealed two TdT mutants—containing either substitution D395N or substitutions D395N+E456N—that possess substantially equalized selectivity toward various dNTPs as compared to the wild-type enzyme. These results will enable rational design of TdT-like enzymes with equalized dNTP selectivity for biotechnological applications.

## 1. Introduction

Terminal deoxynucleotidyl transferase (TdT) is responsible for template-independent random addition of nucleotides to single-stranded DNA during the V(D)J recombination and DNA repair via nonhomologous end joining [1,2]. TdT belongs to the X family of DNA polymerases, alongside polymerases X, β, λ, and μ. Its distinctive feature is the ability to catalyze template-independent DNA synthesis in the 5′→3′ direction; this property is attributed to the length and structure of TdT loop 1 acting as a “complementary base” by interacting with a dNTP and the DNA primer [3]. TdT is capable of catalyzing reactions with various divalent metal ions, such as Mg^2+^, Mn^2+^, and Co^2+^ [4].

TdT is currently widely utilized in biotechnological and molecular biological applications. Its first use was the rapid amplification of cDNA ends (RACE) method demonstrated in the 1980s [5]. In this technique, TdT is used to add a homopolymeric tail to the 3′ end of cDNA, thereby facilitating primer hybridization. Another method involving this enzyme is the TdT-dUTP nick end-labeling (TUNEL) assay [6], which allows us to attach dUTP to 3′ ends of DNA at sites of strand breaks. Subsequently, these sites are visualized with antibodies against dUTP. This approach helps us to detect DNA fragmentation during apoptosis. Nonetheless, one of the most promising applications of TdT is thought to be chemical–enzymatic synthesis of DNA oligonucleotides. The synthesis of a desired DNA sequence from 3′-protected dNTPs has already been demonstrated, e.g., from 3′-O-(2-nitrobenzyl)-2′-deoxyribonucleoside triphosphates [7] or benzoyl/pivaloyl derivatives [8]. In 2018, DNA synthesis using conjugates of TdT and dNTPs was reported, where the enzyme can attach a nucleotide to a primer followed by the detachment of the TdT molecule [9]. Besides, TdT has found applications in more exotic areas such as the development of DNA origami and nanostructures, DNA biosensors, DNA-based information storage, and aptamer synthesis [10].

Nevertheless, a major limitation of all the aforementioned methods is the pronounced selectivity of TdT toward various dNTPs. For human TdT, in the presence of Mg^2+^ ions, the selectivity can be ranked as dGTP > dTTP ≈ dATP > dCTP [4]. By means of an analysis of X-ray crystallographic structures of TdT from *Mus musculus* [3], alignment of TdT orthologs [11], and molecular dynamics (MD) simulations of pre- and post-catalytic complexes of wild-type human TdT [4], amino acid residues Asp395 and Glu456 have been found to be potentially capable of stable interaction with the nitrogenous base of a nucleotide being inserted. Therefore, these residues may play an important role in the dNTP selectivity. Unfortunately, residue Asp395 is poorly resolved in crystal structures mimicking pre- or post-catalytic complexes between TdT and single-stranded DNA primers. In complexes of TdT with double-stranded DNA, Asp395 is engaged in interactions either with the 5′-OH group of a 3′-terminal nucleotide of a template strand (PDB ID 5D46) or with the 3′-flanking guanine base of the template strand (PDB ID 5D49) [12]. During alanine scanning, it was found that substitution of D395A leads to a slight reduction in polymerase activity, and dGTP incorporation occurred more slowly as compared to the three other nucleotides [13]. All data obtained up to now indicate the involvement of Asp395 in the substrate selectivity of TdT. The other residue, Glu456, is less well studied; however, it is known that positional homologs of Glu456 in the PolX family of enzymes interact with the base of an incoming dNTP [14], but the type of the residue at this position varies widely across the entire PolX family [11]. Therefore, the objective of this study was to determine the role of amino acid residues of the active site and loop 1 in the catalytic activity of TdT by in silico mutagenesis and to assess the impact of relevant mutations through MD simulations.

## 2. Results and Discussion

**Figure 1:**
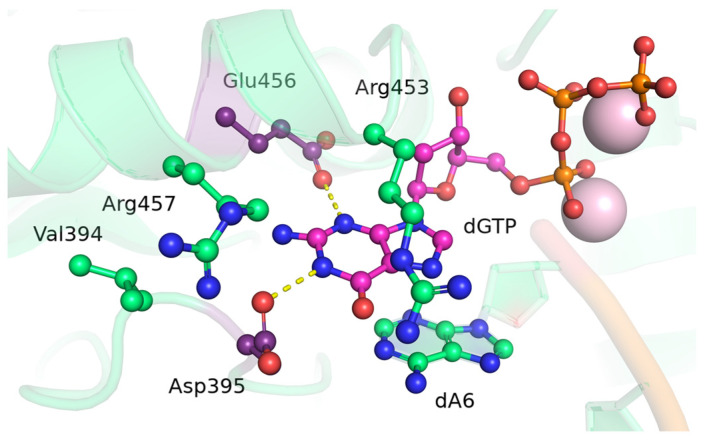
Structure of the active site in the precatalytic complex of TdT with dGTP. Residues forming the most stable hydrogen bonds (yellow dashed lines) with the nitrogenous base of dGTP (pink) are highlighted in violet.

In our previous report [4], through experimental analysis of the purified wild-type enzyme and its mutants, it was found that substitutions of Asp395 and Glu456 in human TdT significantly influence the enzymatic activity. Notably, an analysis of MD trajectories of the wild-type-protein–DNA–dNTP–Mg^2+^ complex in that work revealed stable hydrogen bonds between dGTP (the most readily incorporable dNTP) and these two residues (Figure 1). Therefore, to assess the influence of substitutions of Asp395 and Glu456 on the selection of dNTPs and enzymatic activity, in the present study, we performed detailed in silico analysis.

Aspartic and glutamic acids, owing to their side-chain functional groups, can act only as acceptors of hydrogen bonds. Therefore, it was hypothesized that replacing them with asparagine or glutamine (which are similar in size but capable of acting as acceptors and donors of hydrogen bonds) would lead to changes in the enzyme’s selectivity. In addition, positional substitution D395E was found in approximately a quarter of all analyzed vertebrate species [11]. Unique substitutions have also been found: D395N in the amphibian *Ambystoma mexicanum* and D395K+E456Q in the fish *Cottoperca gobio*, respectively. Therefore, a set of single-substitution and double-substitution mutants of human TdT was designed for in silico analysis: D395E, D395N, D395Q, E456N, E456Q, D395N+E456N, D395Q+E456Q, D395Q+E456N, and D395K+E456Q.

### 2.1. Substitution of Asp395 with Negatively Charged Amino Acid Residue Glu

**Figure 2:**
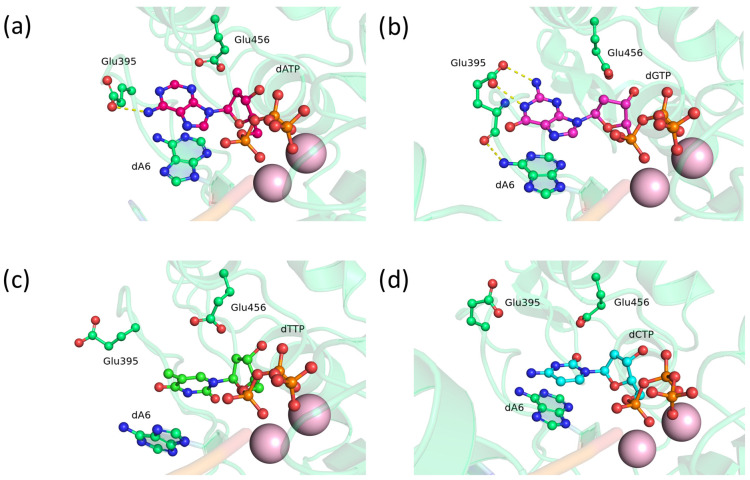
Precatalytic complexes of TdT D395E with (**a**) dATP, (**b**) dGTP, (**c**) dTTP, or (**d**) dCTP. Side chains of amino acid residues 395 and 456 and residues forming hydrogen bonds with them are visualized. Hydrogen bonds are depicted with yellow dashed lines.

Substitution D395E—found in approximately one-quarter of vertebrate species [11]—did not yield results distinguishable from the wild type. In the precatalytic complex of TdT, the Glu395 residue was found to form stable hydrogen bonds with the adenine amino group (Figure 2a) and the N1 atom and exocyclic amino group of guanine (Figure 2b), while not interacting with pyrimidine bases (Figure 2c,d). This outcome was expected because pyrimidines are less stabilized by stacking interactions, with thymine primarily acting as a hydrogen bond acceptor through atoms O_2_ and O_4_.

### 2.2. Substitution of Asp395 with Uncharged Amino Acid Residues Asn and Gln

**Figure 3:**
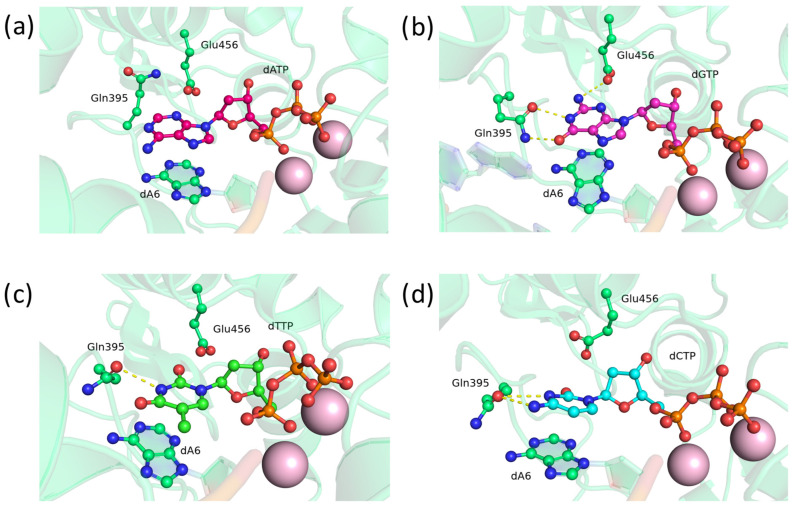
Precatalytic complexes of TdT D395Q with (**a**) dATP, (**b**) dGTP, (**c**) dTTP, or (**d**) dCTP. Side chains of amino acid residues 395 and 456 and residues forming hydrogen bonds with them are visualized. Hydrogen bonds are indicated by yellow dashed lines.

Next, we focused on asparagine and glutamine substitutions at position 395. First, substitutions of Asp395 with Asn and Gln were modeled and analyzed. In the precatalytic complex of TdT D395Q, residue Gln395 forms bonds with atoms N1 and O6 of guanine (Figure 3b), N3 of thymine (Figure 3c), or the N3 atom and amino group of cytosine (Figure 3d). Additionally, stable flipped positionings of Gln395 exist, where the residue does not have any hydrogen bonds with the nitrogenous base of adenosine (Figure 3a). Nevertheless, the D395Q mutant, as anticipated, is capable of entering into hydrogen bonds with nitrogenous bases as an acceptor and donor.

**Figure 4:**
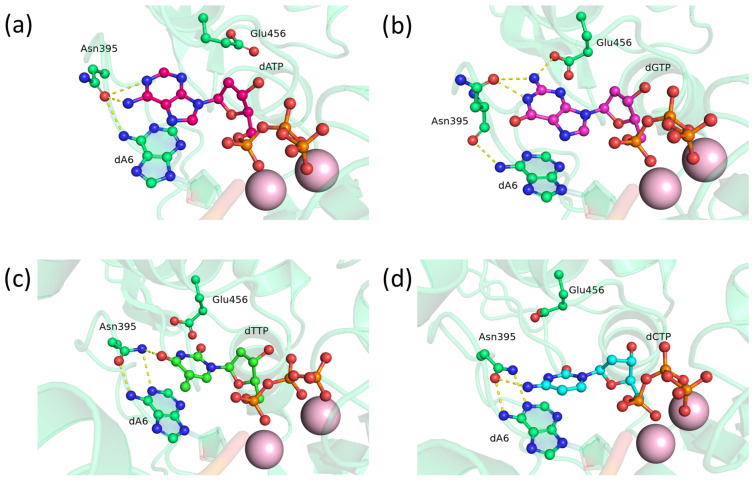
Precatalytic complexes of TdT D395N with (**a**) dATP, (**b**) dGTP, (**c**) dTTP, or (**d**) dCTP. Side chains of amino acid residues 395 and 456 and residues forming hydrogen bonds with them are visualized. Hydrogen bonds are indicated by yellow dashed lines.

In the precatalytic complex of TdT D395N, the Asn395 residue has bonds with the N1 atom and amino group of adenine (Figure 4a), the N1 atom and amino group of guanine (Figure 4b), N3 and O4 of thymine (Figure 4c), or with the amino group of cytosine (Figure 4d). Apparently, due to the smaller size of the asparagine side chain, it is less prone to flipping and breaking hydrogen bonds with the nitrogenous bases as compared to glutamine. Besides, asparagine forms hydrogen bonds with the 3′-terminal adenine through the oxygen of the peptide bond and the amide group, and this arrangement may further stabilize the relative positioning of the nucleotide being embedded and the 3′-terminal nucleotide. Substitution of D395N gave slightly better results than substitution of D395Q, probably because of the difference in the size of their side chains.

### 2.3. Glu456 Substitution with Uncharged Amino Acid Residues Gln and Asn

**Figure 5:**
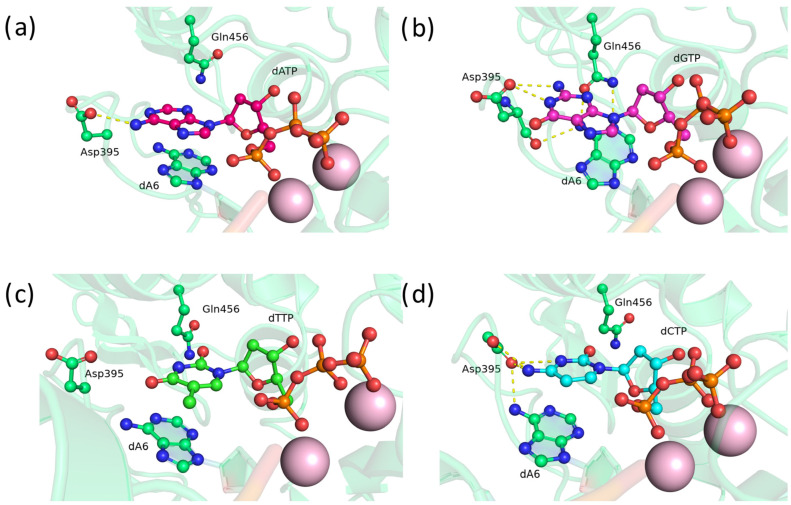
Precatalytic complexes of TdT E456Q with (**a**) dATP, (**b**) dGTP, (**c**) dTTP, or (**d**) dCTP. Side chains of amino acid residues 395 and 456 and the residues forming hydrogen bonds with them are visualized. Hydrogen bonds are shown as yellow dashed lines.

Next, an in silico mutational analysis of glutamic acid at position 456 was conducted. After replacement of Glu456 with Gln in the precatalytic complex, residue Gln456 appears to be too bulky and does not rotate toward the nitrogenous bases, resulting in minimal hydrogen bonding with the bases (Figure 5a,c,d), except for guanine, which is the most easily incorporable (Figure 5b). The original Asp395 formed hydrogen bonds with adenine, guanine, or cytosine as acceptors (Figure 5a,b,d) while also interacting with the 3′-terminal adenine (Figure 5b,c).

**Figure 6:**
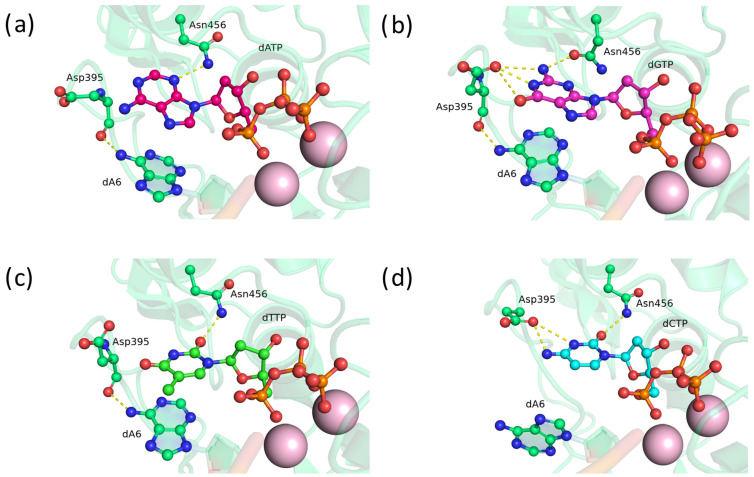
Precatalytic complexes of TdT E456N with (**a**) dATP, (**b**) dGTP, (**c**) dTTP, or (**d**) dCTP. Side chains of amino acid residues 395 and 456 and the residues engaged in hydrogen bonds with them are visualized. Hydrogen bonds are shown as yellow dashed lines.

For asparagine mutant E456N, in the precatalytic complex, residue Asn456 has bonds with the N_3_ atom of adenine (Figure 6a), the amino group of guanine (Figure 6b), O_2_ of thymine (Figure 6c), or O_2_ of cytosine (Figure 6d). Unlike glutamine, asparagine, owing to its smaller size, is capable of easily rotating its side chain and entering into stable hydrogen bonds with all types of DNA nitrogenous bases. For position 456, the difference in the side-chain size proved to be more critical. This effect may be attributed to the fact that residue 456 is located in a stable α-helix, while residue 395 is in unstructured loop 1. Nevertheless, for both positions 395 and 456, substitution with asparagine is more conducive to equalized dNTP selectivity.

### 2.4. The Double Substitution of Asp395 and Glu456

Because of the data on the effects of stand-alone substitutions of Asp395 and Glu456 with glutamines or asparagines, several double substitutions became interesting: D395K+E456Q, D395Q+E456Q, D395Q+E456N, and D395N+E456N. We hypothesized that the combined effect of two substitutions might be greater than the effect of single ones.

**Figure 7:**
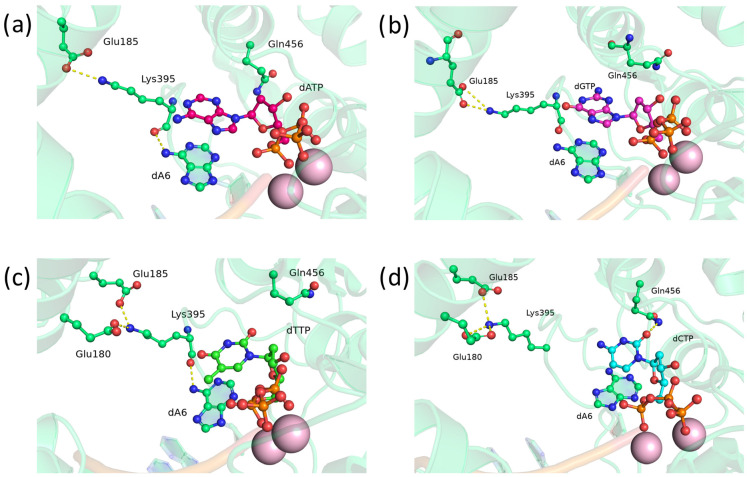
Precatalytic complexes of TdT D395K+E456Q with (**a**) dATP, (**b**) dGTP, (**c**) dTTP, or (**d**) dCTP. Side chains of amino acid residues 395 and 456 and residues forming hydrogen bonds with them are visualized. Hydrogen bonds are presented as yellow dashed lines.

First, mutant D395K+E456Q was analyzed (the substitutions are analogous to those in the TdT of the fish *C. gobio*; Figure 7). Unlike the wild type, where Glu395 forms hydrogen bonds with the nitrogenous base of an incoming nucleotide, the side chain of Lys395 is turned out, thus engaging in stable interactions with Glu180 and Glu185 (Figure 7). Such reorientation should potentially stabilize the positioning of loop 1 (which is responsible for template-independent properties of the enzyme) by means of an additional ionic bridge with the protein backbone. Nevertheless, due to the oxygen atom in the peptide bond, in some MD simulations, Lys395 started to occasionally form hydrogen bonds with the amino group of the adenosine that is located at the 3′ end of the DNA primer (Figure 7a–c).

Residue Gln456 appeared to be excessively bulky and rarely tilted toward a dNTP. Nevertheless, in some cases, hydrogen bond formation between the side chain of Gln456 and the base of a dNTP was observed (Figure 7b,d). Moreover, Gln456 acted as an acceptor with dGTP (Figure 7b) and as a donor with dCTP (Figure 7d).

**Figure 8:**
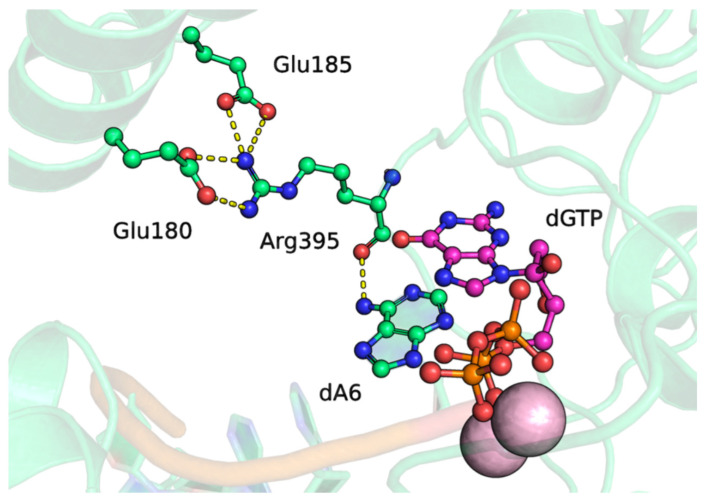
The precatalytic complex of TdT D395R with dGTP. Residues forming hydrogen bonds are visualized. Hydrogen bonds are indicated by yellow dashed lines.

After that, we analyzed the potential ability of a positively charged amino acid residue at position 395 to stabilize the positioning of loop 1. For this purpose, we modeled the complex of the D395R mutant with dGTP, which is the most easily attachable (Figure 8). Indeed, similarly to lysine, residue Arg395 entered into a hydrogen bond with the amino group of the 3′-terminal adenosine via the oxygen of the peptide bond. Additionally, Arg395 engaged in a greater number of interactions with residues Glu180 and Glu185. Therefore, the arginine residue at position 395 may stabilize loop 1 more effectively than lysine can.

Thus, it can be concluded that contacts between loop 1 and the rest of the protein may be important for maintaining the catalytically competent state of the complex of TdT with dNTP and DNA. On the other hand, besides the correct positioning of loop 1 for catalysis, stabilization of the incoming dNTP by hydrogen bonds between it and the protein is necessary, as evidenced by the lower activity of D395K+E456Q compared to the wild type [11].

**Figure 9:**
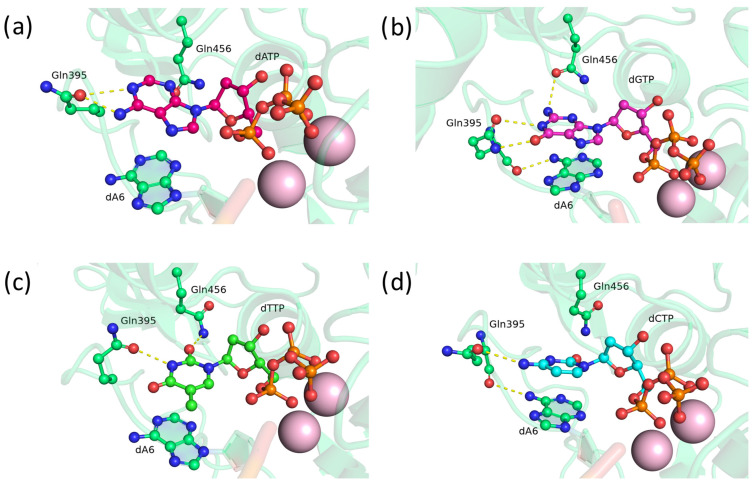
Precatalytic complexes of TdT D395Q+E456Q with (**a**) dATP, (**b**) dGTP, (**c**) dTTP, or (**d**) dCTP. Side chains of amino acid residues 395 and 456 and the residues forming hydrogen bonds with them are visualized. Hydrogen bonds are shown as yellow dashed lines.

In the precatalytic complex of double mutant TdT D395Q+E456Q, residue Gln395 formed stable hydrogen bonds with the N1 atom and amino group of adenine (Figure 9a), O_6_ and N_1_ of guanine (Figure 9b), the N_3_ atom of thymine (Figure 9c), or the amino group of cytosine (Figure 9d). Furthermore, Gln395 was capable of entering into a hydrogen bond with the amino group of the 3′-terminal adenine through the oxygen of the peptide bond (Figure 9b,d). Despite its size, residue Gln456 had hydrogen bonds with the amino group of guanine (Figure 9b) or the O_2_ atom of thymine (Figure 9c). Apparently, the presence of two glutamine residues at positions 395 and 456 increased the chances of both residues’ forming hydrogen bonds with deoxynucleotide analogs, thereby contributing to a stabler positioning of the nucleotide in the active site of TdT.

**Figure 10:**
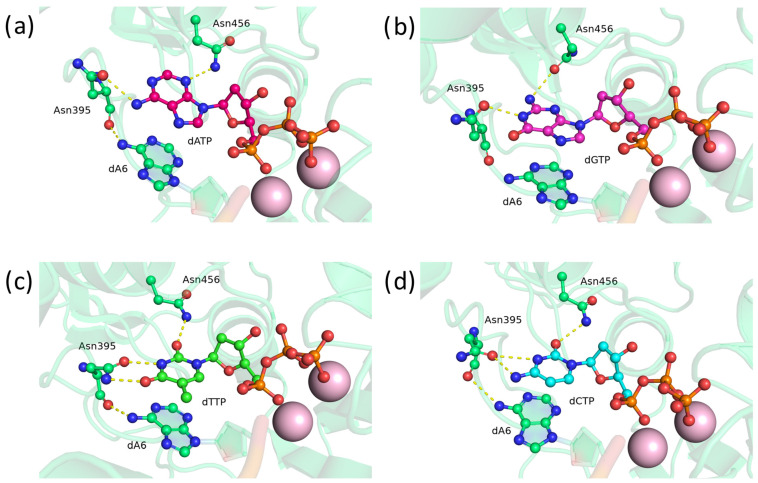
Precatalytic complexes of TdT D395N+E456N with (**a**) dATP, (**b**) dGTP, (**c**) dTTP, or (**d**) dCTP. Side chains of amino acid residues 395 and 456 and residues forming hydrogen bonds with them are visualized. Hydrogen bonds are indicated by yellow dashed lines.

Of all nine mutations potentially altering substrate selectivity of human terminal deoxynucleotidyl transferase, the most interesting result was obtained with the double asparagine substitution in mutant D395N+E456N (Figure 10). The asparagine residue was small enough to freely turn toward the dNTP at the 456th position, while not engaging in stable interactions with the protein backbone at the 395th position. Additionally, the amide group in the asparagine side chain can act not only as a hydrogen bond acceptor (Figure 10b) but also as a donor (Figure 10a,d) owing to the nitrogen atom.

Residue Asn395 formed stable hydrogen bonds with all types of nitrogenous bases of dNTP: with the adenine amino group (Figure 10a), the N1 atom of guanine (Figure 10b), N_3_ and O_4_ of thymine (Figure 10c), or the N_3_ atom and amino group of cytosine (Figure 10d). Moreover, Asn395 is able to form two hydrogen bonds with nitrogenous bases (Figure 10b,d). In addition, Asn395 frequently interacts with the 3′-terminal adenine of the DNA primer (Figure 10a,c,d).

Residue Asn456 almost always turned toward dNTP and formed a stable hydrogen bond with it as an acceptor in case of dGTP and dCTP (Figure 10b,d), or as a donor with dATP and dTTP (Figure 10a,c). Asn456 entered into bonds with N_3_ of adenine (Figure 10a), the amino group of guanine (Figure 10b), or the O_2_ atom of thymine or cytosine (Figure 10c,d). The formation of two hydrogen bonds with the nitrogenous base was not observed for Asn456.

On the basis of the findings about paired glutamine and asparagine substitutions, we hypothesized that residue 395 should be replaced by Gln to form stabler bonds with pyrimidine nucleotides, while residue 456 should be replaced by shorter Asn to more likely rotate toward a dNTP. Accordingly, mutant D395Q+E456N was simulated and analyzed.

**Figure 11:**
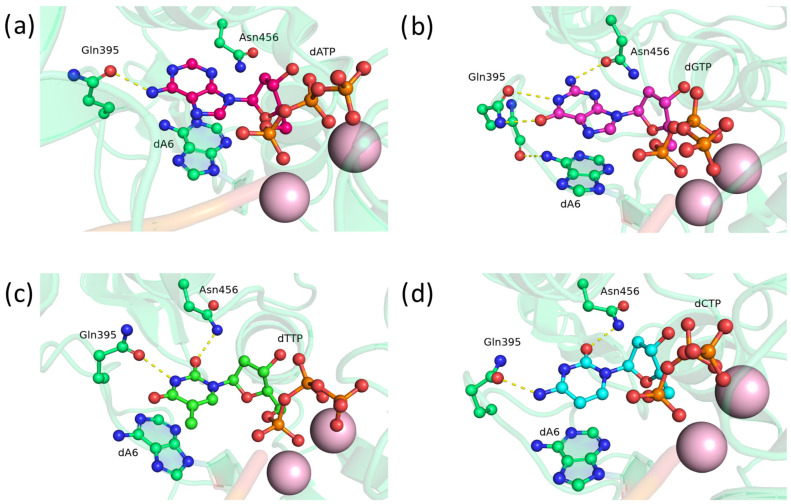
Precatalytic complexes of TdT D395Q+E456N with (**a**) dATP, (**b**) dGTP, (**c**) dTTP, or (**d**) dCTP. Side chains of amino acid residues 395 and 456 and residues forming hydrogen bonds with them are visualized. Hydrogen bonds are indicated by yellow dashed lines.

Residue Gln395 formed bonds with the amino group of adenine (Figure 11a), N1 and O6 atoms of guanine (Figure 11b), N3 of thymine (Figure 11c), or the amino group of cytosine (Figure 11d). Similarly to the single mutant, Gln395 is capable of forming a bond with the amino group of the 3′-terminal adenine (Figure 11b). Residue Asn456 entered into hydrogen bonds with the amino group of guanine (Figure 11b) or the O_2_ atom of thymine or cytosine (Figure 11c,d). Thus, we demonstrated that the proposed amino acid substitutions in principle can form hydrogen bonds with the nitrogenous base of dNTPs.

### 2.5. Average Number of Hydrogen Bonds between the Protein and the Nucleobase of an Incoming dNTP

Previously [4], using the MMPBSA approach for free-energy estimates of pre- and post-catalytic complexes formed by the wild-type enzyme, it was found that due to the highly charged nature of both ligands and protein-active sites, free-energy estimates have significant fluctuations and thereby are a very inconclusive parameter for characterizing the stability of the modeled complexes. At the same time, the number of hydrogen bonds between the active site residues and the nucleobase as well as H-bond lifetime parameter, which together provided the average number of H-bonds within the simulation trajectory, revealed correlations with the experimental results. Thereby, this parameter was selected as the in silico properties predictor within the present study of mutant forms of the enzyme.

**Figure 12:**
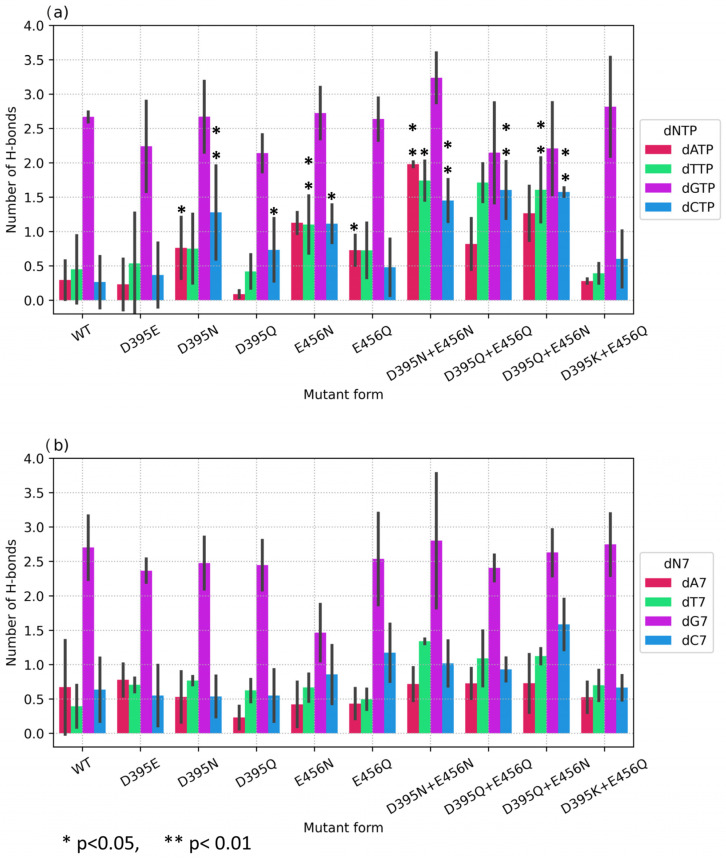
Average numbers of hydrogen bonds between dNTP or 3′-terminal nucleotide and amino acid residues of the protein for (**a**) a precatalytic complex and (**b**) a post-catalytic complex. Mutant enzymes are plotted on the X-axis. The average number of hydrogen bonds for three or more independent replicates of each complex is plotted on the Y-axis. Error bars represent the standard deviation among replicates.

To assess the quantitative contribution of the proposed substitutions, the average number of hydrogen bonds between the protein and the base of an embedded or 3′-terminal nucleotide was evaluated for pre- and post-catalytic complexes, respectively (Figure 12). First of all, one can clearly see the huge excess (more than 5-fold) of hydrogen bonds formed by the wild-type enzyme with dGTP as compared to the other nucleotides. Nevertheless, other substantial differences in the H-bond network were documented for some mutants in their precatalytic complexes (Figure 12a). For instance, for single mutant D395N interacting with dATP, the number of hydrogen bonds was 0.76 compared to 0.29 for the wild type, and with dCTP, the number of hydrogen bonds was 1.28 compared to 0.26 for the wild type. Mutant D395Q also manifested increased dCTP selectivity: the number of bonds with dCTP was 0.73 compared to 0.26 for the wild type, but no significant differences were found for complexes with other dNTPs. On average, E456N formed 1.13 hydrogen bonds with dATP, and 1.12 with dCTP. From this finding, it can be concluded that single asparagine substitutions D395N and E456N yielded better results as compared to glutamine substitutions D456Q and E456Q.

Much more pronounced equalization of dNTP selectivity was achieved with all double mutants, which showed an increase in the number of H-bonds with dATP, dCTP, and dTTP, but not dGTP (Figure 12a). Statistically significant differences were found for most mutants, except for natural variant D395K+E456Q, when compared with the wild-type enzyme. For instance, for D395Q+E456Q, the average number of hydrogen bonds with dCTP was 1.61. Double substitution D395Q+E456N caused an increase in the average number of hydrogen bonds with dATP up to 1.27, and for dCTP up to 1.58. Mutant D395N+E456N showed the most statistically significant differences for all three deoxynucleotides by achieving 1.98 H-bonds with dATP, 1.45 with dCTP, and 1.74 with dTTP.

Notably, for post-catalytic complexes, no significant differences were found, indicating a lower tendency of residues 395 and 456 to form hydrogen bonds with an already embedded nucleotide. The decrease in stability of the positioning of the 3′-terminal nucleotide in the active site is necessary for the enzyme to transition to the next catalytic action and to avoid substrate inhibition.

Additionally, it could be noted that enzyme efficacy depends not only on the amino acid residues conformation and contacts, but also conformation of the 2′-deoxyribose moiety of 3′-terminal nucleotide in the primer. The crystal structures of catalytic intermediates suggest that complex formation happens with 3′-terminal primer 2′-deoxyribose in C2′-endo conformation and a shift to C3′-endo conformation occurs prior to the catalytic step. The simulations revealed that the 3′-terminal nucleotide 2′-deoxyribose samples both C3′-endo and C2′-endo, as well as other states, like O4′-endo. However, current molecular dynamics force fields are known to disfavor C3′-endo conformation in DNA [15,16], even in simulations of protein–DNA complexes where contact with the protein enforces the C3′-endo conformation [17]. While our simulations reveal some effects on the conformation of the 2′-deoxyribose moiety of 3′-terminal nucleotide, explicitly considering them would be a dubious effort due to aforementioned force-field deficiencies.

### 2.6. Average Number of Hydrogen Bonds between the Protein and the Nucleobase of a 3′-Terminal Nucleotide

**Figure 13:**
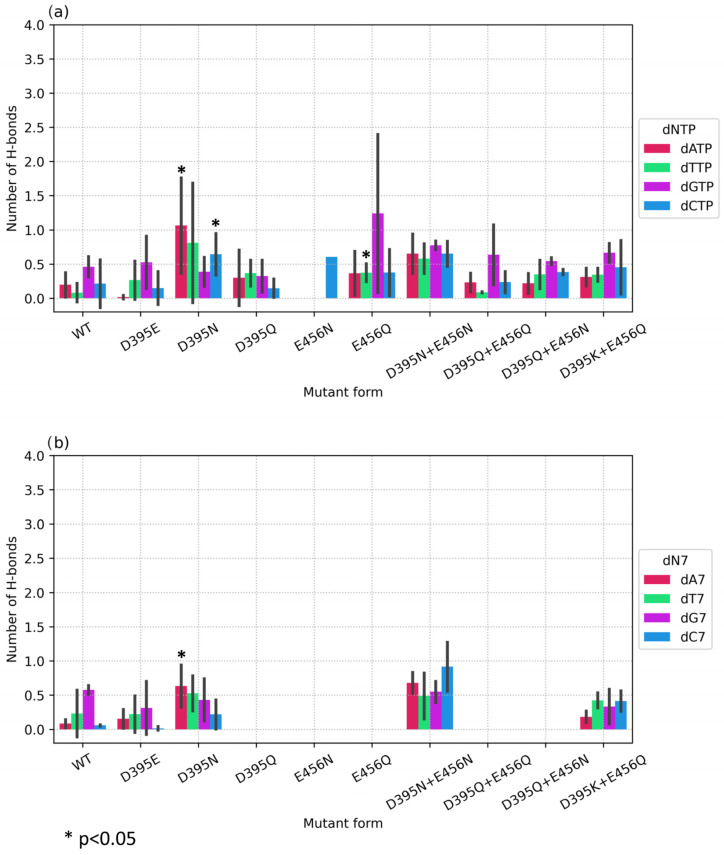
The average number of hydrogen bonds between the 3′-terminal nucleotide and amino acid residues in a pre- (**a**) or post-catalytic (**b**) complex.

Another necessary condition for the catalytic reaction to proceed is a proper mutual arrangement of planes of nitrogenous bases of an incoming dNTP and 3′-terminal nucleotide. Therefore, hydrogen bonds with the 3′-terminal nucleotide (adenosine in the simulated models) were calculated for pre- and post-catalytic complexes (Figure 13).

It should be noted that total number of hydrogen bonds with a 3′-terminal nucleotide was much lower compared with the incoming dNTP. Nevertheless, statistically significant increases in the number of hydrogen bonds for the precatalytic complex were registered for D395N with dATP and dCTP (Figure 13a) and for D395N+E456N with dTTP (Figure 13a). From this result, it can be concluded that substitution D395N leads to an increase both in the number of hydrogen bonds with the base of an embedded dNTP and in the number of hydrogen bonds with the 3′-terminal adenine. Moreover, in post-catalytic complexes of D395N and of D395N+E456N, an increased level of H-bonding was found, too (Figure 13b), indicating a greater role of amino acid residue 395—compared to position 456—in the formation of hydrogen bonds with both dNTP and DNA termini.

## 3. Materials and Methods

### 3.1. The Protein Model

Pre- and post-catalytic complexes of human TdT were examined by homology modeling with Modeller [18] by means of X-ray structures of mouse TdT complexes representing pre- and post-catalytic states [3] as well as time-resolved X-ray structures of a catalytic complex of human TdT [19].

The initial structures of pre- and post-catalytic complexes were constructed for the wild-type enzyme and for corresponding mutants (D395E, D395Q, D395N, E456Q, E456N, D395Q+E456Q, D395Q+E456N, D395N+E456N, and D395K+E456Q) in complex with either hexameric DNA (5′-GGAAGA-3′) and four different dNTPs or heptameric DNA (5′-GGAAGAN-3′) and various 3′-terminal nucleotides, respectively. Additionally, an elongated complex was modeled for pyrimidine nucleotides: mutant D396N+E456N interacting with either dTTP and 5′-GGAAGT-3′ or dCTP and 5′-GGAAGC-3′.

### 3.2. Modeling Parameters

MD simulations were carried out using the GROMACS software package [20] with AMBER force-field parameters version 14SB for the protein [21] and OL15 for DNA [22]. The TIP3P water model was employed with respective ion parameters [23,24].

Parameterization of dNTPs was performed similarly [25], utilizing the RED server for charge optimization [26,27,28]. Magnesium ions in the enzyme’s active site were described via a distributed charge model [29]. Protonation states of protein amino acid residues were determined from data contained within the literature and stored on the H++ server [30].

Energy minimization was performed until a maximum force threshold of 500 kJ/(mol·nm) was reached, with a total of 50,000 steps and a step size of emstep = 0.01. After energy minimization, two subsequent equilibrations were conducted in NVT and NPT ensembles, respectively. The parameters for the NVT ensemble were a total time of 1 ns (dt = 0.002 ps, n-steps = 500,000), a V-rescale thermostat with equilibration and velocity generation temperatures set to 300 K, and constraints on hydrogen bond vibrations applied by the LINCS method.

For the NPT ensemble, the parameters were as follows: a total time of 1 ns (dt = 0.002 ps, n-steps = 500,000), the V-rescale thermostat [31], the Parrinello–Rahman barostat [32], with constraints on hydrogen bond vibrations applied by the LINCS method.

The MD simulations were conducted with the same parameters as in the NPT ensemble, except with a duration of 100 ns (dt = 0.002 ps, n-steps = 50,000,000). At least three replicates were obtained for each model.

### 3.3. Data Processing

Data extraction from MD trajectories was performed with the help of standard functions of the GROMACS software. Compilation of summary tables was carried out via bash scripting commands. Python 3.10 programming language with numpy, pandas, matplotlib.pyplot, seaborn, and scipy.stats modules was utilized for graph plotting and statistical analysis. The Shapiro–Wilk test was employed to determine the statistical significance.

Representative frames from molecular trajectories were selected using clustering of frames based on the nitrogenous base of a nucleotide being inserted or the 3′-terminal nucleotide for pre- and post-catalytic complexes, respectively, as well as amino acid residues of the active site. The threshold for the root mean square deviation used for clustering was set to 0.13 nm.

## 4. Conclusions

A detailed analysis of the molecular interactions that ensure substrate selectivity of human terminal deoxynucleotidyl transferase was carried out in this study. MD models of pre- and post-catalytic complexes were obtained for the wild-type enzyme and a set of single-substitution and double-substitution mutants. MD simulations of mutants featuring substitution D395E, D395Q, D395N, E456Q, or E456N or double substitution D395Q+E456Q, D395Q+E456N, or D395N+E456N enabled us to deeply analyze the interactions of this enzyme with each dNTP and to find key amino acid residues participating in the dNTP selection. It was found that stabilization of the nucleobase of an incoming dNTP by an H-bond network may be crucial for dNTP selectivity. Among all tested mutants of TdT, the formation of an additional hydrogen bond in the precatalytic complex between amino acid residues and dATP, dTTP, or dCTP was observed for the mutants carrying substitution D395N or E456N. Moreover, two additional hydrogen bonds were found in cases of double mutants D395Q+E456Q, D395Q+E456N, and D395N+E456N. It was shown that TdT mutant D395N+E456N forms the most stable hydrogen bonds between the introduced asparagine residues and each dNTP.

Finally, it can be concluded that we successfully analyzed the factors that alter the dNTP selectivity of human terminal deoxynucleotidyl transferase, and these results will facilitate the rational design of TdT-like enzymes with equalized dNTP selectivity for biotechnological applications.

## Data Availability

Data are available from N.A.K. and T.E.T. upon request. Tel.: +7-(383)-363-5174, e-mail: nikita.kuznetsov@niboch.nsc.ru (N.A.K.); tyugashev@niboch.nsc.ru (T.E.T.).
